# Performance of GPT-3.5 and GPT-4 on the Korean Pharmacist Licensing Examination: Comparison Study

**DOI:** 10.2196/57451

**Published:** 2024-12-04

**Authors:** Hye Kyung Jin, EunYoung Kim

**Affiliations:** 1 Research Institute of Pharmaceutical Sciences, College of Pharmacy, Chung-Ang University, Seoul, Republic of Korea; 2 Data Science, Evidence-Based and Clinical Research Laboratory, Department of Health, Social, and Clinical Pharmacy, College of Pharmacy, Chung-Ang University, Seoul, Republic of Korea; 3 Division of Licensing of Medicines and Regulatory Science, The Graduate School of Pharmaceutical Management and Regulatory Science Policy, The Graduate School of Pharmaceutical Regulatory Sciences, Chung-Ang University, 84 Heukseok-Ro, Dongjak-gu, Seoul, 06974, Republic of Korea, 82 2-820-5791, 82 2-816-7338

**Keywords:** GPT-3.5, GPT-4, Korean, Korean Pharmacist Licensing Examination, KPLE

## Abstract

**Background:**

ChatGPT, a recently developed artificial intelligence chatbot and a notable large language model, has demonstrated improved performance on medical field examinations. However, there is currently little research on its efficacy in languages other than English or in pharmacy-related examinations.

**Objective:**

This study aimed to evaluate the performance of GPT models on the Korean Pharmacist Licensing Examination (KPLE).

**Methods:**

We evaluated the percentage of correct answers provided by 2 different versions of ChatGPT (GPT-3.5 and GPT-4) for all multiple-choice single-answer KPLE questions, excluding image-based questions. In total, 320, 317, and 323 questions from the 2021, 2022, and 2023 KPLEs, respectively, were included in the final analysis, which consisted of 4 units: Biopharmacy, Industrial Pharmacy, Clinical and Practical Pharmacy, and Medical Health Legislation.

**Results:**

The 3-year average percentage of correct answers was 86.5% (830/960) for GPT-4 and 60.7% (583/960) for GPT-3.5. GPT model accuracy was highest in Biopharmacy (GPT-3.5 77/96, 80.2% in 2022; GPT-4 87/90, 96.7% in 2021) and lowest in Medical Health Legislation (GPT-3.5 8/20, 40% in 2022; GPT-4 12/20, 60% in 2022). Additionally, when comparing the performance of artificial intelligence with that of human participants, pharmacy students outperformed GPT-3.5 but not GPT-4.

**Conclusions:**

In the last 3 years, GPT models have performed very close to or exceeded the passing threshold for the KPLE. This study demonstrates the potential of large language models in the pharmacy domain; however, extensive research is needed to evaluate their reliability and ensure their secure application in pharmacy contexts due to several inherent challenges. Addressing these limitations could make GPT models more effective auxiliary tools for pharmacy education.

## Introduction

Recently, artificial intelligence (AI) based on large language models (LLMs) has shown promise in various fields and industries [1-3]. On November 30, 2022, ChatGPT (GPT-3.5), an AI language model trained using deep-learning algorithms, was released by OpenAI [4]. Since its release, ChatGPT has become a popular topic, showing promise in optimizing performance on various examinations, such as the US Certified Public Accountant examination [5] and those in MBA and law school programs [6,7]. ChatGPT has also demonstrated potential efficacy in the health care field, such as in optimizing clinical workflows and supporting clinical decisions and diagnoses [8-11]. Furthermore, it has performed adequately in medical education, with demonstrated effectiveness on 6 different national medical licensing examinations, including those in Italy, France, Spain, the United States, India, and the United Kingdom [12,13]. These findings indicate the potential of ChatGPT as an innovative method for medical education and as a study resource, with efficient and accurate responses [14,15].

ChatGPT’s demonstrated ability to perform well on medical and licensing examinations suggests that this technology could also be applicable to other health care–related examinations. In Japan, ChatGPT’s performance on the National Nurse Examinations from 2019 to 2023 showed an average accuracy over 5 years of 75.1% for basic knowledge questions and 64.5% for general questions, with the passing criteria being 80% and approximately 60%, respectively [16]. In addition, ChatGPT achieved response accuracy rates between 54.1%-63.8% across 1510 questions on Taiwan’s registered nurse license examination [17]. Similarly, for dentistry questions via the Swiss Federal Licensing Examination in Dental Medicine, it showed an average accuracy rate of 63.3% [18].

Compared with its predecessor GPT-3.5, the proficiency of GPT-4 in responding to the United States Medical Licensing Examination (USMLE) questions showed an accuracy of 90.7% across the entire USMLE, which surpassed the passing threshold of approximately 60% accuracy [19]. Furthermore, while GPT-3.5 scored 42.8% on the National Medical Licensing Examination in Japan, GPT-4 achieved a score of 81.5%, surpassing the passing threshold of 72% [20]. However, despite their high performance in medical education, the utility of these programs has yet to be extensively studied in the context of pharmacy education. In particular, research on their performance on national pharmacist licensing examinations is limited, with Nisar et al [21] being one of the few relevant studies. Their study demonstrated that ChatGPT can achieve satisfactory accuracy and relevance when responding to pharmacology textbook queries related to pharmacokinetics, clinical applications, adverse effects, and drug interactions, suggesting that the application of LLMs in the pharmacy domain is increasingly viable. However, the study also highlighted the need for further improvements in ChatGPT’s performance when addressing more intricate and complex questions. This indicates that while promising, the technology still requires refinement for broader clinical applications. Given that pharmacists are responsible for providing comprehensive drug information and suggesting personalized treatment plans for patients, the performance of AI technologies like ChatGPT that can assist in these tasks must be evaluated.

This study aims to investigate the accuracy of GPT-3.5 and GPT-4 on the Korean Pharmacist Licensing Examinations (KPLEs) conducted from 2021 to 2023. Responses from the KPLEs were used to conduct a comparative analysis of ChatGPT’s performance across various units.

## Methods

### ChatGPT Models

In this study, we assessed the performance of 2 versions of an AI model: GPT-3.5 and GPT-4 (a newer, paid version available through the ChatGPT+ platform). We accessed these models through the online interface provided on OpenAI’s website rather than the application programming interface.

### KPLE Datasets

We used the original questions from the 72nd, 73rd, and 74th KPLEs, respectively held in 2021, 2022, and 2023. These examinations are conducted annually and comprise 350 questions classified into 4 units: Biopharmacy (100 questions), which includes biochemistry, molecular biology, microbiology, immunology, pharmacology, preventive pharmacy, and pathophysiology; Industrial Pharmacy (90 questions), covering areas such as physical pharmacy, synthetic chemistry, medicinal chemistry, pharmaceutical analysis, pharmaceutics, pharmacognosy, and herbal medicine; Clinical and Practical Pharmacy, divided into part I (77 questions) focusing on pharmacotherapy and part II (63 questions) covering pharmacy practice, pharmaceutical manufacturing, pharmaceutical quality control, and pharmacy administration and management; and Medical Health Legislation (20 questions), which includes Pharmaceutical Affairs Act, Narcotics Control Act, National Health Promotion Act, Framework Act on Health and Medical Services, National Health Insurance Act, and Regional Public Health Act. Each question is worth 1 point. The passing criteria for the KPLE requires a minimum score of 40% or higher for each subject and a total score of 60% or higher, equating to at least 210 points across all subjects.

### Procedures

The questions from the KPLEs, along with their multiple-choice responses, were utilized in their Korean format, in conjunction with the official national examination guidelines. Image-based questions that ChatGPT could not recognize were excluded when calculating accuracy. Specifically, 30, 33, and 27 questions from 2021, 2022, and 2023 were excluded from the final analysis. To elicit diverse responses, we provided specific instructions via a prompt stating, “Only one best option can be selected.” The determination of “correct” responses to the inquiries posed to GPT-3.5 and GPT-4 was grounded in the Korea Health Personnel Licensing Examination Institute’s (KHPLEI) database and accessed from their official website [22]. Only responses that strictly adhered to the question instructions were considered “correct.” Across all 3 years, the most image-based questions that were excluded came from Unit 2, Industrial Pharmacy, ranging from 20 (22.2%) to 24 (26.7%) of the 90 questions. Additionally, for non–image-based questions included in tables, we redescribed the information in a manner similar to how a test administrator or proctor reads examination questions for visually impaired students (ie, those who are blind or have low vision). Responses that were ambiguous, contained clear errors, or for which ChatGPT generated multiple answers with incorrect options instead of a single response were considered inaccurate. We provided no additional content or hints in our study, aiming to simulate a real examination scenario. However, we followed up by asking, “Do you have confidence in this?” to assess the model’s stability and consistency and to prompt ChatGPT’s potential re-evaluation of its initial response. However, these data were not included in the results and were kept for reference purposes only. If the model’s answer changed, it could suggest a degree of doubt in its initial response. Monitoring how frequently and under what conditions the model adjusts its answers provides important information about its capacity for self-correction, which is crucial for learning and decision-making. We collected and evaluated responses from GPT-3.5 and GPT-4 on December 17 and 18, 2023, according to these criteria for correctness.

### Data Analysis

We employed standard descriptive statistics including numbers, proportions, and averages for each dataset. A Fisher exact test was utilized to compare the rates of correct responses. Statistical analyses were performed using SPSS software, version 29 (IBM Corp). All tests were 2-tailed, and a *P* value of less than .05 was considered statistically significant.

### Ethics Approval

Ethical approval was not required for this study because it involved the analysis of data from a publicly available database. The test questions and answers used in this study were initially developed and copyrighted by the KHPLEI and are available for academic research purposes. The KHPLEI holds all copyrights pertaining to the examination content and ensured that this research complied with these copyrights without violation.

## Results

### ChatGPT’s Performance

Out of a total of 350 questions each, 320, 317, and 323 questions from the 2021, 2022, and 2023 KPLEs, respectively, were included in the final analysis. The 3-year average percentage of correct answers from GPT-3.5 and GPT-4 were 60.7% (583/960) and 86.5% (830/960), respectively. When analyzing accuracy rates by unit, the GPT models showed their most notable performance over the 3-year period in Biopharmacy. Specifically, GPT-4 achieved a 96.7% (87/90) accuracy rate in 2021, while GPT-3.5 recorded a lower but still impressive accuracy rate of 80.2% (77/96) in 2022. In contrast, the accuracy rates were lowest in Medical Health Legislation out of the 4 units. The lowest scores were observed in 2022, with accuracy rates of 40% (8/20) and 60% (12/20) for GPT-3.5 and GPT-4, respectively.

Beyond our numerical analysis, we found that GPT-4 provided more comprehensive and accurate explanations for its responses compared to its predecessors.

In the 2021 KPLE, 320 (91.4%) of the 350 questions were suitable for analysis in both GPT-3.5 and GPT-4, excluding 30 image-based questions. Of these, GPT-3.5 correctly answered 200 questions, resulting in an accuracy rate of 62.5%. In contrast, GPT-4 answered 275 questions correctly, achieving an accuracy rate of 85.9%. Table 1 presents the detailed scores for each unit of the 2021 KPLE. Regarding specific question types, GPT-4 notably surpassed GPT-3.5 in achieving higher rates of correct responses across all sections, with statistically significant differences (all *P*<.05) observed in all comparisons except for Unit 4. The highest accuracy rates were in Biopharmacy (GPT-3.5 66/90, 73.3%; GPT-4 87/90, 96.7%), while the lowest were in Medical Health Legislation (GPT-3.5 10/20, 50%; GPT-4 13/20, 65%).

**Table 1.** Comparison of GPT-3.5’s and GPT-4’s performances on the 2021 Korean Pharmacist Licensing Examination.

Question category	All questions, n	Student correct response rate, n (%)	Questions answerable by GPT, n	GPT-3.5 correct response rate, n (%)	GPT-4 correct response rate, n (%)	Passing criteria, %	*P* value
Total	350	246 (70.3)	320	200 (62.5)	275 (85.9)	≥60	<.001
Unit 1: Biopharmacy^a^	100	70.3 (70.3)	90	66 (73.3)	87 (96.7)	≥40	<.001
Unit 2: Industrial Pharmacy^b^	90	60.2 (66.9)	70	39 (55.7)	55 (78.6)	≥40	.007
Unit 3: Clinical and Practical Pharmacy I and II^c^	140	100.2 (71.6)	140	85 (60.7)	120 (85.7)	≥40	<.001
Unit 4: Medical Health Legislation^d^	20	15.3 (76.5)	20	10 (50)	13 (65)	≥40	.52

a Biochemistry, molecular biology, microbiology, immunology, pharmacology, preventive pharmacy, and pathophysiology.

b Physical pharmacy, synthetic chemistry, medicinal chemistry, pharmaceutical analysis, pharmaceutics, pharmacognosy, and herbal medicine.

c Pharmacotherapy, pharmacy practice, pharmaceutical manufacturing, pharmaceutical quality control, and pharmacy administration and management.

d Pharmaceutical Affairs Act, Narcotics Control Act, National Health Promotion Act, Framework Act on Health and Medical Services, National Health Insurance Act, and Regional Public Health Act.

Out of 350 questions on the 2022 KPLE, 317 (90.6%) were analyzed by both GPT-3.5 and GPT-4, and the correct response rates are shown in Table 2. Neither version could process 33 image-based questions. GPT-3.5 correctly answered 188 of the 317 questions, resulting in a 59.3% accuracy rate, falling short of the required 60% standard for passing. In contrast, GPT-4 answered 273 of the 317 questions correctly, achieving an accuracy rate of 86.1%. Regarding question types, the highest accuracy rates were observed in Biopharmacy, at 80.2% (77/96) and 95.8% (92/96) for GPT-3.5 and GPT-4, respectively. In contrast, the lowest accuracy rates were recorded for Medical Health Legislation, with GPT-3.5 and GPT-4 achieving 40% (8/20) and 60% (12/20), respectively. Notably, GPT-4 showed a considerable 40.9% increase in accuracy for Industrial Pharmacy questions compared with GPT-3.5 (*P*<.001).

**Table 2.** Comparison of GPT-3.5’s and GPT-4’s performances on the 2022 Korean Pharmacist Licensing Examination.

Question category	All questions, n	Student correct response rate, n (%)	Questions answerable by GPT, n	GPT-3.5 correct response rate, n (%)	GPT-4 correct response rate, n (%)	Passing criteria, %	*P* value
Total	350	248 (70.9)	317	188 (59.3)	273 (86.1)	≥60	<.001
Unit 1: Biopharmacy^a^	100	67 (67)	96	77 (80.2)	92 (95.8)	≥40	.001
Unit 2: Industrial Pharmacy^b^	90	62.8 (69.8)	66	32 (48.5)	59 (89.4)	≥40	<.001
Unit 3: Clinical and Practical Pharmacy I and II^c^	140	101.7 (72.6)	135	71 (52.6)	110 (81.5)	≥40	<.001
Unit 4: Medical Health Legislation^d^	20	16.5 (82.5)	20	8 (40)	12 (60)	≥40	.34

a Biochemistry, molecular biology, microbiology, immunology, pharmacology, preventive pharmacy, and pathophysiology.

b Physical pharmacy, synthetic chemistry, medicinal chemistry, pharmaceutical analysis, pharmaceutics, pharmacognosy, and herbal medicine.

c Pharmacotherapy, pharmacy practice, pharmaceutical manufacturing, pharmaceutical quality control, and pharmacy administration and management.

d Pharmaceutical Affairs Act, Narcotics Control Act, National Health Promotion Act, Framework Act on Health and Medical Services, National Health Insurance Act, and Regional Public Health Act.

As shown in Table 3, 323 out of 350 (92.3%) questions on the 2023 KPLE were answered by both GPT-3.5 and GPT-4, with 27 image-based questions that could not be processed. GPT-4 substantially exceeded GPT-3.5 in correct response rates across all question types, with statistically significant differences (*P*<.001) in all categories except for Unit 4. The highest accuracy rates were in Biopharmacy (GPT-3.5 72/99, 72.7%; GPT-4 93/99, 93.9%), whereas the lowest rates were in Industrial Pharmacy for GPT-3.5 (31/66, 47%) and Medical Health Legislation for GPT-4 (14/20, 70%).

**Table 3.** Comparison of GPT-3.5’s and GPT-4’s performances on the 2023 Korean Pharmacist Licensing Examination.

Question category	All questions, n	Student correct response rate, n (%)	Questions answerable by GPT, n	GPT-3.5 correct response rate, n (%)	GPT-4 correct response rate, n (%)	Passing criteria, %	*P* value
Total	350	257.3 (73.5)	323	195 (60.4)	282 (87.3)	≥60	<.001
Unit 1: Biopharmacy^a^	100	74.7 (74.7)	99	72 (72.7)	93 (93.9)	≥40	<.001
Unit 2: Industrial Pharmacy^b^	90	63.8 (70.9)	66	31 (47)	53 (80.3)	≥40	<.001
Unit 3: Clinical and Practical Pharmacy I and II^c^	140	103 (73.6)	138	81 (58.7)	122 (88.4)	≥40	<.001
Unit 4: Medical Health Legislation^d^	20	15.7 (78.5)	20	11 (55)	14 (70)	≥40	.51

a Biochemistry, molecular biology, microbiology, immunology, pharmacology, preventive pharmacy, and pathophysiology.

b Physical pharmacy, synthetic chemistry, medicinal chemistry, pharmaceutical analysis, pharmaceutics, pharmacognosy, and herbal medicine.

c Pharmacotherapy, pharmacy practice, pharmaceutical manufacturing, pharmaceutical quality control, and pharmacy administration and management.

d Pharmaceutical Affairs Act, Narcotics Control Act, National Health Promotion Act, Framework Act on Health and Medical Services, National Health Insurance Act, and Regional Public Health Act.

### GPT Models Versus Humans

KPLE participants totaled 1920, 1993, and 2014 in 2021, 2022, and 2023, respectively. For the 2021 examination, 59.7% (1147/1920) of the participants were female, with the largest age group being those in their 20s (1205/1920, 62.8%). This trend continued in 2022 and 2023, with women comprising 59% (1175/1993 in 2022; 1188/2014 in 2023) of the total participants in both years, and those in their 20s comprising 58.6% (1167/1993) and 59.2% (1193/2014), respectively. All participants were either graduates or expected graduates of a pharmacy school, as this level of education is required to qualify for the examination [23-26].

**Figure 1.**
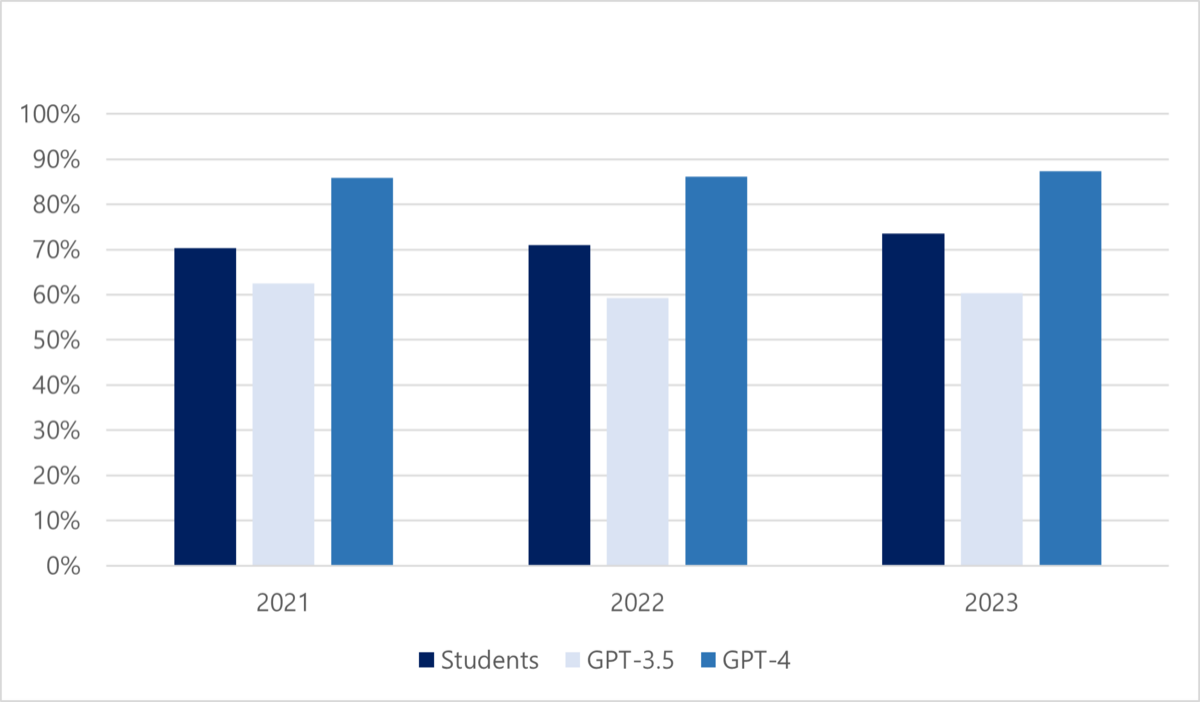
Performance of GPT-3.5, GPT-4, and pharmacy students on the Korean Pharmacist Licensing Examination.

When comparing AI and human performance, pharmacy students correctly answered an average of 70.3%, 70.9%, and 73.5% of the same questions as those used in our study, in 2021, 2022, and 2023, respectively, with an average passing rate of 92.3%. GPT-3.5 showed lower accuracy rates than human participants, with scores of 62.5% (200/320), 59.3% (188/317), and 60.4% (195/323), respectively, whereas GPT-4 demonstrated higher accuracy rates of 85.9% (275/320), 86.1% (273/317), and 87.3% (282/323), respectively (Figure 1).

## Discussion

### Principal Findings

This study evaluated the performance of both GPT-3.5 and GPT-4 on the KPLE over 3 recent years. GPT-4 consistently achieved scores above the passing level, whereas GPT-3.5 did not reach the 60% passing threshold in 1 of the 3 years, instead scoring very close to this criteria at 59.3% (188/317), indicating the limitations of GPT-3.5 in answering KPLE questions accurately. In contrast, GPT-4 exhibited significantly improved correct response rates across all units compared to GPT-3.5, surpassing the passing threshold with an overall accuracy of 86.5% (830/960). In evaluations of the performance of AI compared with that of human participants, pharmacy students’ accuracy rates were higher than those of GPT-3.5 but lower than those of GPT-4. This aligns with a recent study comparing medical students’ knowledge and interpretation-based responses with those of GPT-3.5, wherein GPT-3.5 achieved an accuracy rate of 60.8% (48/79). This was lower than the students’ overall performance, with an average accuracy rate of approximately 90.8% (71.8/79) [27].

The findings of GPT-3.5’s underperformance in comparison to GPT-4 are consistent with those of a previous study that investigated the performance of GPT-3 and GPT-4 in the North American Pharmacist Licensure Examination [28] as well as in other health professional examinations [20,29,30]. In addition, similar studies have shown that GPT-3.5 failed to pass such examinations, such as the Taiwanese Pharmacist Licensing Examination [31]. This could be attributed to GPT-3.5’s training data cutoff in September 2021, rendering it outdated in terms of recent pharmacy practice advancements, research, and guideline updates. Moreover, disparity in performance might be attributed to differences in language and culture, as well as variations in the content of the examinations [32]. Both GPT models have acquired substantial information on health care policies in English-speaking nations because of their comprehensive English datasets. Similarly, previous studies have reported higher accuracy rates for questions in English when compared to other languages [31,33,34]. Consequently, these findings imply that GPT models might benefit from supplementary training data in languages besides English, to expand their knowledge and enhance their performance on language-specific pharmacy examinations. These findings suggest that LLMs possess great capabilities in addressing KPLE questions, and with further refinement, these models can be reasonably expected to provide even higher levels of accuracy. This aligns with recent research in medical natural language processing, which has increasingly emphasized the importance of domain- and language-specific modeling [35,36]. Models specialized in specific languages, such as Korean, and the medical domain have shown superior performance in tasks like processing medical documents compared to general models, highlighting the need for the continued development of AI models tailored to specific domains and languages [35,36].

Notably, among the 4 units, the accuracy rate was highest for Biopharmacy questions among the specific question datasets used in our study, followed by Clinical and Practical Pharmacy. This suggests that GPT-4 could potentially support pharmacists within clinical settings. However, this may not accurately reflect the complexity of real-world clinical settings, which involve a variety of patient cases and collaborations with other health care professionals. Additionally, the KPLE does not involve communication in the pharmacist-patient relationship, interpersonal skills, or empathy. Brin et al [37] found that GPT-4 exhibited superior performance in soft skills such as empathy, ethics, and judgment. This finding indicates AI’s potential to address complex ethical challenges, show empathy, and effectively support patient care and family interactions. However, that study had a limited question pool of only 80 multiple-choice questions drawn from 2 sources, potentially leading to selection bias. Consequently, the questions might not accurately reflect the full scope of the actual USMLE content or cover all the soft skills that are vital in medical practice. Furthermore, ChatGPT models scored the lowest in Medical Health Legislation, which may be attributable to the differences in policies and laws between Korea and the United States, as the AI is likely more familiar with legal frameworks in English-speaking countries.

Additionally, GPT models are susceptible to a phenomenon termed “hallucination,” where they generate scientifically inaccurate information that seems plausible to individuals lacking expertise [34]. For example, in our study, GPT-4 generated an incorrect drug interaction between 2 medications that are not actually known to interact. In another example, GPT-3.5 suggested an incorrect dosage for a common medication, which could lead to potential harm if used in a clinical setting. Moreover, GPT-3.5 showed lower concordance and higher self-contradiction compared to GPT-4. These examples highlight the importance of critically evaluating AI-generated information, especially in the context of clinical environment. Depending solely on generated content carries risks; therefore, those receiving the output should have the professional pharmaceutical knowledge necessary to assess its accuracy. These limitations must be recognized and addressed to comprehensively evaluate GPT-4’s practical applicability in classrooms and clinical practice.

Previous studies suggested that GPT models have the potential to become useful tools in the field of medical education because of their ability to generate appropriate and precise information in response to well-defined inputs [12,38,39]. Due to their ease of access and rapid information generation capabilities, AI-based LLMs, like ChatGPT, are poised to serve as valuable educational aids. Our evaluation findings align with these studies and indicate that GPT models could facilitate a “self-directed” learning approach, helping students enhance their knowledge and reasoning skills. However, it is essential to validate the information provided by GPT models because they lack standard references for the retrieved information. Hence, with careful implementation, clear protocols, and oversight by health care professionals, AI-driven chatbots show significant potential to transform clinical pharmacology and drug information services. Specific guidelines, such as ensuring chatbot use is supervised by licensed professionals and continuously updated with the latest medical knowledge, are crucial for their effective and safe application.

### Limitations

Our study has some limitations. First, it is essential to recognize that the GPT models employed in this study may not reflect the latest models available. Therefore, caution should be exercised when implementing these findings in practical clinical settings, since depending on up-to-date references is essential. Specifically, the findings represent the GPT models’ capabilities up to December 18, 2023, and variations in results may occur in the future, given the expected rapid improvement in the capabilities of ChatGPT versions through user feedback and deep learning. Additional updates are expected in the future, and it is crucial to consistently assess them. Second, image-based test questions were excluded because the AI models could not support them. Moreover, the AI chatbots were unable to interpret the information presented in tables; therefore, we manually entered this information. Third, the findings of this study are specific to the datasets and conditions of the KPLE. However, the adjustments noted above were randomly distributed across various subjects and not biased toward any specific topics. Furthermore, as both GPT-3.5 and GPT-4 were evaluated under the same conditions, these exclusions likely had minimal impact on the final performance comparison. However, the generalizability of these results to other professional environments or licensing examinations may still be limited. Further research is needed to assess the performance of AI chatbots in different contexts and with various datasets, as it remains possible that correct answers may not be obtained under different conditions. Finally, it should be noted that this investigation focused exclusively on GPT-3.5 and GPT-4. In the future, it is worth considering the possibility of implementing other LLMs, such as New Bing and Bard, in the pharmaceutical field.

### Conclusions

Our study demonstrates the potential of ChatGPT (ie, GPT-3.5 and GPT-4) to assist in pharmaceutical knowledge comprehension within the Korean context. GPT-4 exhibited expert-level performance and consistently passed the KPLE, while GPT-3.5 fell short of the passing criteria in 1 instance. Pharmacy students outperformed GPT-3.5 but scored lower than GPT-4. Although GPT-4 outperformed students, educators and students should not rely solely on chatbots for learning, as AI tools may produce misleading or inaccurate information. Therefore, it is imperative to conduct thorough testing and validation before successfully implementing AI and to examine the feasibility of all GPT versions in real-world clinical contexts. Future research should incorporate more extensive and diverse question sets and ethical scenarios to provide a more accurate representation of pharmacy practice.

## Abbreviations

AI: artificial intelligence
KHPLEI: Korea Health Personnel Licensing Examination Institute
KPLE: Korean Pharmacist Licensing Examination
LLM: large language model
USMLE: United States Medical Licensing Examination
